# An analysis of SPECT/CT non-visualization of sentinel lymph nodes in renal tumors

**DOI:** 10.1186/s13550-018-0460-y

**Published:** 2018-12-03

**Authors:** Teele Kuusk, Maarten L. Donswijk, Renato A. Valdés Olmos, Roderick E. De Bruijn, Oscar R. Brouwer, Kees Hendricksen, Simon Horenblas, Katarzyna Jóźwiak, Warner Prevoo, Henk G. Van Der Poel, Bas W. G. Van Rhijn, Esther M. Wit, Axel Bex

**Affiliations:** 1 Department of Urology, The Netherlands Cancer Institute, Postbus 90203, 1006 BE Amsterdam, The Netherlands; 2 Department of Nuclear Medicine, The Netherlands Cancer Institute, Postbus 90203, 1006 BE Amsterdam, The Netherlands; 3 Department of Radiology, Interventional Molecular Imaging Laboratory and Nuclear Medicine Section, Leiden University Medical Center, P.O. Box 9600, 2300 RC Leiden, The Netherlands; 4 Department of Epidemiology and Biostatistics, The Netherlands Cancer Institute, Postbus 90203, 1006 BE Amsterdam, The Netherlands; 5 Department of Radiology, The Netherlands Cancer Institute, Postbus 90203, 1006 BE Amsterdam, The Netherlands; 6 Division of Surgical Oncology, Department of Urology, The Netherlands Cancer Institute, Plesmanlaan 121, 1066 CX Amsterdam, The Netherlands

**Keywords:** Detection failure, Lymphoscintigraphy, Nanocolloid, Renal cell carcinoma, SPECT/CT, Sentinel lymph node

## Abstract

**Background:**

Sentinel lymph node biopsy (SLNB) after intratumoral injection of ^99m^Tc labeled nanocolloid and imaging with scintigraphy and SPECT/CT in renal tumors is feasible. However, sentinel lymph node (SN) non-detection rate with scintigraphy and SPECT/CT is high. The aim of the study was to determine factors affecting non-visualization (NV) of SN imaging in renal tumors. Seventy-eight patients with cT1–3 renal tumors received intratumoral injection of 225 MBq ^99m^Tc-labeled nanocolloid 1 day before (partial) nephrectomy. Radiotracer injection was followed by anterioposterior and lateral scintigraphy in combination with SPECT/CT 20 min and 2–4 h after. Surgical treatment of the tumor with sentinel lymph node biopsy by aid of γ-probe and-camera was performed the next day. Scintigraphy and SPECT/CT images were evaluated and patient, tumor, and procedure characteristics were collected for 73 eligible patients used in uni- and multivariable analysis of a potential association with NV.

**Results:**

A total of 80 (mean 1.1, IQR 0–2, max 6) sentinel lymph nodes in 46 patients were detected with scintigraphy and SPECT/CT. Preoperative visualization rate and intraoperative detection rate was 63% [95% CI 50–73%] and 61% [95% CI 49–72%], respectively. In uni- and multivariable analysis, the only factor associated with non-visualization was age, showing higher odds of non-visualization with higher age.

**Conclusion:**

Our study demonstrated that non-visualization of SNs in renal tumors is relatively high and is associated with patient age. Furthermore, kidneys and also its tumors are highly vascularized which may cause a wash-out effect that could be identified with decreased kidney-liver ratios. However, in our data, the effect was statistically inconclusive. Further studies are needed to improve visualization and standardize the procedure of SLNB in renal tumors. The percentage of NV limits the use of SLNB for research and clinical purposes in renal cancer.

**Electronic supplementary material:**

The online version of this article (10.1186/s13550-018-0460-y) contains supplementary material, which is available to authorized users.

## Background

Despite low incidence of lymph node (LN) metastases in renal cancer (RCC), dissemination into lymph nodes portends extremely poor prognosis [1, 2]. In low risk and high risk cancers, the detection rate with a routine pathohistological staining is only 4% and 10%, respectively and lymph node dissection (LND) in low risk cancers has no proven survival benefit [3, 4]. Furthermore, lymphatic drainage in renal tumors is unpredictable and drainage outside local retroperitoneal lymph nodes may be one of the reasons for the lack of a survival advantage with conventional LND [5–7]. Sentinel lymph node biopsy (SLNB) typically consists of a preoperative lymphoscintigraphy to indicate the anatomical location of the first tumor-draining lymph nodes, i.e., the sentinel node(s), combined with a γ-probe guided surgical biopsy of these nodes. SLNB may improve staging of RCC especially in patients who have aberrant drainage within or outside retroperitoneal lumbar lymph node basins and might have a role in future translational research of tumor immunology and biology of early metastasis. It has been suggested in prostate cancer that extended pelvic lymph node dissection combined with SLNB increases the yield of nodal lymph node metastases, especially in high risk disease [8]. Due to proven efficacy in staging procedures in many cancer types and less associated morbidity of SLNB compared to LND, SLNB is routinely used in melanoma, breast, head-neck, vulvar, cervical, and penile cancer and actively studied in many other tumor types [9, 10]. Feasibility of SLNB procedure in renal tumors has been studied and confirmed earlier by our group and others [11, 12]. However, both groups had high rate of non-visualization of sentinel lymph node (SN) on scintigraphy and single-photon emission computed tomography with computed tomography (SPECT/CT). Also, the procedure is currently lacking standardization. These factors may hamper widespread applicability and adoption to further study its use in clinical practice. Therefore, the aim of our study was to determine incidence and predictive factors of non-visualization of SN on scintigraphy and SPECT/CT in renal tumors, evaluate detection rate at surgery, and propose a standardized protocol for future studies.

## Methods

### Patients

**Fig. 1:**
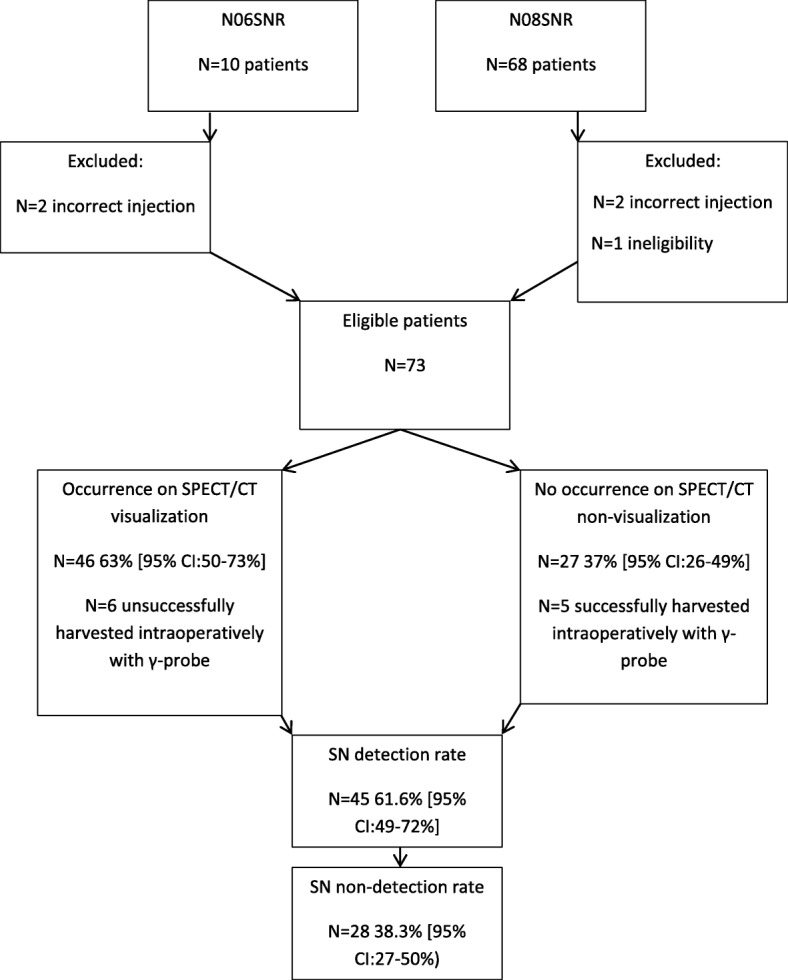
Consort diagram for study participants

From 2008 to 2017, 78 patients were enrolled into a feasibility and a single-arm phase II prospective study to investigate lymphatic drainage and the distribution of SN in renal tumors (N06SNR and N08SNR registered under NL26406.031.08 Fig. 1). The studies had medical ethics committee approval and all patients signed written informed consent. Inclusion criteria were CT-based cT1–3 renal tumors ≤ 10 cm of any subtype, and clinically and radiologically non-metastatic disease (cN0cM0), assessed with pelvic, abdominal, and thoracic contrast-enhanced CT, age > 18 years, life expectancy > 3 months, WHO performance status 0–1, and no prior systemic therapy. Primary endpoint was the percentage of SNs located at any site outside the left or right locoregional retroperitoneal template (LRT) on lymphoscintigraphy and subsequent SPECT/CT imaging as described below. The sample size of the phase II study was based on a Simon two-stage design including 40 patients with SN on SPECT/CT imaging in the final analysis. Patients without visualization of SN were recorded to analyze the failure rate. For this analysis, our objective was to assess the rate and factors contributing to non-visualization in both studies.

### Sentinel node imaging

One day prior to surgery, a dose of approximately 225 MBq of ^99m^Tc nanocolloid (Nanocoll©; GE Healthcare, Eindhoven, Netherlands) in a volume of 0.4 ml was injected percutaneously under ultrasound or CT guidance into the tumor. Primary tumors ≤ 4 cm were injected centrally with a volume of 0.4 ml and 4–10 cm tumors were injected with 2–3 depots of 0.4 ml around the center avoiding necrotic areas. Following injection, lymphoscintigraphy based on anterioposterior and lateral 5-min planar static images after 20 min and 2–4 h was acquired. Subsequently, SPECT/CT was acquired using a hybrid system (SymbiaT, Siemens, Erlangen, Germany). After correction for scatter and tissue attenuation, SPECT and CT images were fused. Multiplanar reconstruction enabled comparison of fused images with concomitant CT images to determine the anatomical location of the SNs (Osirix Dicom viewer with medical imaging software, Pixmeo, Geneva, Switzerland). The nodes draining directly from the tumor on planar lymphoscintigraphy were considered to be SNs and were localized using SPECT/CT. In case of multiple visualized lymph nodes in a basin, the early appearing nodes were considered to be the SNs. Aspects related to tracer injection and activity (spillage, anterior/posterior injection, number of depots, properly injected depot according to SPECT/CT image, kidney/liver activity, depot location) were prospectively evaluated from lymphoscintigraphy and SPECT/CT images by an experienced nuclear medicine physician. Data on injected radiotracer dosage, number of injections, and modality of injection (US or CT) were collected retrospectively from patients’ electronic files. All other factors as described in Tables 1 and 2 were retrieved from medical records.

**Table 1:** Factors for visualization, univariable and multivariable analysis

Factor	Univariable analysis		Multivariable analysis	
	OR [95% CI]	*P* value	OR [95% CI]	*P* value
Number of patients	73			
Age (years)	0.92 [0.87–0.97]	*0*.*008*	0.88 [0.81–0.95]	*0*.*002*
Age above 59 (years)				
Yes	1.0			
No	0.44 [0.15–1.22]	0.11		
Gender				
Female	1.0			
Male	0.85 [0.32–2.20]	0.74		
BMI	0.99 [0.92–1.06]	0.99		
Tumor size (cm)	0.89 [0.71–1.12]	0.12		
Side				
Right	1.0			
Left	0.92 [0.35–2.40]	0.87		
Polarity				
Upper pole	1.0			
Intermedial pole	2.35 [0.53–10.4]	0.25		
Lower pole	0.71 [0.24–2.09]	0.53		
Tumor location				
Posterior				
Yes	1.0			
No	0.65 [0.25–1.70]	0.38		
Renal score				
Low	1.0			
Moderate	0.75 [0.20–2.77]	0.66		
High	0.84 [0.27–2.62]	0.76		
pT stage				
T1a	1.0			
T1b	2.7 [0.33–21.9]	0.35		
T2a	0.91 [0.19–4.35]	0.91		
T2b	0.60 [0.08–4.40]	0.61		
T3a	1.20 [0.07–19.6]	0.89		
ccRCC				
Yes	1.0			
No	0.42 [0.13–1.33]	0.14		
Non ccRCC				
Yes	1.46 [0.44–4.86]	0.53		
No	1.0			
Lymphovascular invasion				
Yes	1.0			
No	1.19 [0.20–6.97]	0.84		
Necrosis on imaging				
Yes	1.0			
No	0.41 [0.10–1.72]	0.22		
Necrosis in histology				
Yes	1.0		1.0	
No	0.42 [0.16–1.12]	0.08	1.63 [0.44–6.05]	0.46
Leibovich score				
Low	1.0			
Moderate	0.65 [0.16–2.60]	0.54		
High	0.52 [0.12–2.18]	0.37		
High risk				
Yes	0.58 [0.13–2.65]	0.48		
No	1.0			
Dose of isotope	0.99 [0.97–1.01]	0.83		
Number of injections	1.47 [0.57–3.80]	0.46		
Spillage				
Yes	1.0			
No	1.84 [0.51–6.55]	0.34		
Depot properly injected				
Yes	1.0			
No	0.96 [0.35–2.61]	0.93		
Anterior depot				
Yes	1.0			
No	1.15 [0.38–3.45]	0.79		
Time between injection and early imaging	1.0 [1.0–1.0]	0.69		
Time between injection and late imaging	1.0 [1.0–1.0]	0.25		
Kidney/liver activity ratio at early scintigraphy	1.0 [0.99–1.00]	0.08	0.98 [0.95–1.01]	0.45
Kidney/liver activity ratio at late scintigraphy	1.0 [0.99–1.00]	*0*.*05*	1.02 [0.97–1.07]	0.37
Metastases				
Yes	1.0			
No	0.96 [0.30–3.05]	0.96		
Death				
Yes	0.52 [0.22–1.23]	0.13		
No	1.0			

Numbers in italic are statistically significant *p* ≤ 0.05. *BMI* body mass index, *SN* sentinel node, *ccRCC* clear cell renal cell carcinoma, *OR* odds ratio

**Table 2:** Patient and procedure characteristics for visualization and non-visualization

	Visualization *N* and % or median and IQR	Non-visualization *N* and % or median and IQR
Number of patients	46 (63%)	27 (37%)
Gender		
Female	22 (47.8%)	14 (51.9%)
Male	24 (52.2%)	13 (48.1%)
Age, years (median, IQR)	56 (49–63)	62 (54–70)
Age above 59 years	21 (45.7%)	17 (63%)
Age below 59 years	25 (54.3%)	10 (37%)
BMI (median, IQR)	27 (23–30)	26 (24–32)
Size of the tumor (cm) (median, IQR)	5.6 cm (4.1–7.0)	6.5 cm (5.0–8.0)
Tumor location		
Side		
Right	23 (50%)	13 (48.1%)
Left	23 (50%)	14 (51.9%)
Polarity		
Upper pole	12 (26.1%)	3 (11.1%)
Intermedial pole	17 (37%)	14 (51.9%)
Lower pole	17 (37%)	10 (37.1%)
Axial position		
Posterior	19 (41.3%)	14 (51.9%)
Anterior	27 (58.7%)	13 (48.1%)
Renal score		
Low risk	7 (15.2%)	5 (18.5%)
Moderate risk	11 (23.9%)	7 (25.9%)
High risk	28 (60.9%)	15 (55.6%)
pTstage		
T1a	9 (19.6%)	2 (7.4%)
T1b	26 (56.5%)	17 (63%)
T2a	4 (8.7%)	4 (14.8%)
T2b	2 (4.3%)	1 (3.7%)
T3a	5 (10.9%)	3 (11.1%)
pNstage		
N0	44 (95.7%)	21 (77.8%)
N1	1 (2.2%)	1 (3.7%)
Nx	1 (2.2%)	5 (18.5%)
Histology		
ccRCC	30 (65.2%)	22 (81.5%)
Pap I	5 (10.9%)	2 (7.4%)
Pap II	3 (6.5%)	1 (3.7%)
Chromophobe	4 (8.7%)	1 (3.7%)
Oncocytoma	3 (6.5%)	0
Fibrous tumor	1 (2.2%)	0
NOS	0	1 (3.7%)
Fuhrman grade		
I	4 (8.9%)	4 (14.8%)
II	12 (26.7%)	12 (44.4%)
III	13 (28.9%)	3 (11.1%)
IV	0	4 (14.8%)
NA	17 (37.8%)	4 (14.8%)
Leibovich score		
Low	18 (39%)	10 (37%)
Intermediate	13 (28.3%)	9 (33.3%)
High	4 (8.7%)	4 (14.8%)
NA	11 (23.9%)	4 (14.8%)
Lymphovascular invasion		
Yes	4 (8.7%)	2 (7.4%)
No	42 (91.3%)	25 (92.6%)
Necrosis in histology		
Yes	16 (34.8%)	15 (55.6%)
No	30 (65.2%)	12 (44.4%)
Necrosis on imaging		
Yes	4 (8.7%)	5 (18.5%)
No	42 (91.3%)	22 (81.5%)
SN radioactivity detected with γ-probe and camera		
Yes	40 (87.0%)	5 (18%)
No	6 (13.0%)	22 (81.5%)
Injection modality		
UH	45 (97.8%)	27 (100%)
CT	1 (2.2%)	0
^99m^Tc dose, MBq (median, IQR)	209 (187–222)	212 (196–218)
Number of injections		
1 injection	27 (58.7%)	18 (66.7%)
2 injections	18 (39.1%)	9 (33.3%)
3 injections	1 (2.2%)	0
Spillage		
Yes	13 (28.3%)	4 (14.8%)
No	30 (65.2%)	17 (63%)
NA	3 (6.5%)	6 (22.2%)
Depot properly Injected		
Yes	30 (65.2%)	14 (51.9%)
No	2 (4.3%)	1 (3.7%)
NA	14 (30.4%)	12 (44.4%)
Anterior depot	19 (41.3%)	11 (40.7%)
Posterior depot	18 (39.1%)	9 (33.3%)
Not anterior nor posterior	9 (19.6%)	7 (25.9%)
Number of depots		
1	26 (59.1%)	13 (48.1%)
2	17 (38.6%)	5 (18.5%)
3	0	2 (7.4%)
Data not available	3 (6.5%)	7 (25.9%)
SN visualization		
On early planar scintigraphy	5 (10.9%)	NA
On late planar scintigraphy	9 (19.6%)	
On early planar scintigraphy combined with SPECT/CT	32 (69.6%)	
SN number on imaging (mean, IQR, sum)	1.1 (0–2, 80)	NA
SN number harvested at surgery (mean, IQR, sum)	2.1 (0–3, 155)	NA
Non SN number harvested at surgery (mean, IQR, sum)	3.0 (0–4, 220)	NA
Time between injection and 1st imaging in minutes (median, IQR)	28 min (15 min–65 min)	28 min (15 min–1 h 5 min)
Time between injection and 2nd Imaging in minutes (median, IQR)	149 min (175 min–188 min)	182 min (139 min–194 min)
Kidney/liver activity ratio at early scintigraphy (median, IQR)	22.4 (4.0–51.6)	6.6 (1.5–17.6)
Kidney/liver activity ratio at late scintigraphy (median, IQR)	10.7 (3.7–26.9)	3.6 (1.5–11.4)

*BMI* body mass index, *IQR* interquartile range, *SN* sentinel node, *NA* not applicable

### Sentinel lymph node biopsy

The following day, surgical treatment of the primary tumor combined with SN mapping was performed. In both studies, the boundaries of the locoregional templates were defined as described previously [5, 13]. Surgical approach (open, laparoscopic, robot-assisted) was decided per case depending on the primary tumor. At surgery, SNs were located in the areas indicated by preoperative SPECT/CT images and detected intraoperatively with a γ-probe (Neoprobe, Johnson&Johnson Medical, Hamburg, Germany) in combination with a portable γ-camera (Sentinella, S102, GEM imaging, Valencia, Spain). Harvested SNs were also measured ex situ with both γ-probe and γ-camera. After SN excision, the surgical area was scanned using the portable γ-camera to verify complete SN removal. For ethical reasons, only SNs accessible through the chosen surgical approach were removed. Sentinel nodes were formalin fixed, paraffin embedded, and cut into 3 μm sections according to our institute SLNB protocol. Paraffin sections were stained and examined with hematoxylin and eosin.

### Non-sentinel lymph node dissection

Additionally, non-SNs within the retroperitoneal lymph node dissection template were resected for further standard hematoxylin-eosin staining.

### Statistical analysis

Patient and SLNB procedure characteristics were analyzed with descriptive statistics. Factors associated with the SN visualization were analyzed using a logistic regression. The analysis included patient data, such as age, gender, BMI (body mass index); tumor characteristics such as tumor size, pT stage, side, polarity, RENAL score, which categorizes renal masses by complexity for surgical decision making, Leibovich score, which predicts metastases free survival after surgical therapy, tumor posteriorly located (yes/no), histology, lymphovascular invasion, necrosis on imaging, necrosis in histology; and procedural and injection techniques characteristics such as volume of the isotope, number of injections, spillage on imaging, defined by a spillage of the tracer > 25% outside of the tumor seen on SPECT/CT images, depot properly injected, defined as having a depot of the tracer > 75% inside the tumor (yes/no), depot located anteriorly (yes/no), whereas kidney was divided anterior and posterior on coronal plane at the level of hilar vessels, to determine the complexity of injection because injection anteriorly is more challenging, actual time between injection and early scintigraphic imaging, time between injection and late scintigraphic imaging, kidney/liver activity ratio (calculated by drawing a region of interest (ROI) over the depot in the kidney, then dividing the maximum number of counts in this ROI by the maximum number of counts in a second ROI over the liver) at both early and late planar scintigraphy as a potential indicator of wash-out of the tracer; and outcome parameters such as any distant metastases (yes/no), death (yes/no) (Table 1). Additional logistic regression was performed with age categories split at median age of the patients to test whether this cut-off can be used to define a group of patients with a non-visualization. For testing wash-out association with non-visualization, we calculated the ratio (activity in the kidney) / (activity in the liver), whereas higher grades of radiotracer wash-out decrease this ratio. *P* value ≤ 0.05 was considered statistically significant and all tests were two-sided. Odds ratios are presented with their 95% confidence intervals (CI). Factors for which *p* value reached < 0.1 in a univariable analysis were included in a multivariable model. Statistical analyses were performed using SPSS version 22 (IBM, Chicago, IL, USA).

## Results

Of the 78 patients, 5 patients were excluded because of ineligibility. Therefore, 73 patients were available for final analysis (Fig. 1). The characteristics of the patients and the procedure are shown in Table 2. The majority of patients (68.5%) had low or intermediate risk clear cell RCC. Median time between the injection and early and late imaging with visualization and non-visualization was 33 min (IQR 19–51 min), 28 min (IQR 15–65 min) and 149 min (IQR 175–188), 182 min (IQR 139–194) respectively. Median tracer dose was 209 mBq (IQR 187–222) and 212 (IQR 196–218) with visualization and non-visualization, respectively. Visualization of SNs on imaging was 63% [95% CI 50–73%]. All SNs visualized on planar images were also visualized on SPECT/CT. A total of 80 SNs in 46 patients were visualized, and the mean number of SNs on imaging was 1.1 (IQR 0–2).

In six patients, no SN could be harvested. In three patients, there was no activity detected with a γ-probe in vivo nor ex vivo and a selective dissection of lymph nodes was performed in the area of the SNs as visualized on SPECT/CT. In three patients, radioactive SNs were detected with a γ-probe but these were not harvested due to severe obesity and risky dissection or non-accessibility through the exposure. On the contrary, in five patients with non-visualization on pre-operative imaging, radioactive SNs were detected with a γ-probe the next day at surgery. Therefore, SNs were detected and harvested in 45 patients (61% [95% CI 49–72%]). The mean number of harvested SNs was 2.12 (IQR 0–3) and mean number of non-SNs was 3.01 (IQR 0–4). A total of 155 SNs were removed. Two patients had occult SN metastases (one was with visualized SN and the other had non-visualization).

### Factors associated with non-visualization

Table 1 shows factors for visualization, univariable and multivariable analysis. In univariable and multivariable analysis, an increased risk of non-visualization was associated with patient age, showing a trend toward older patients having higher risk of non-visualization (*p* = 0.008 and 0.002, respectively). Whereas including age categories with below and above median age of 59 years did not show statistical significance in univariable analysis (*p* = 0.11). Other factor associated with non-visualization in univariable analysis was kidney/liver activity on scintigraphy at late planar imaging; however, this factor did not reach statistical significance in multivariable model.

## Discussion

SLNB in general has a role in multiple tumors in studying lymphatic drainage as well as having importance in staging with an advantage of being a more sensitive and less aggressive procedure than LND [9, 14–17]. SLNB has proven efficacy in breast, head-neck, vulvar, cervical and penile cancer, and melanoma and is actively studied in many other tumor types [9, 18, 19]. The first feasibility and outcome studies of SLNB in renal tumors have been published showing that the procedure is safe; however, further studies in a larger cohort for clinical or research utility in high risk cancer may be limited due to a significant rate of non-visualization. Two groups that studied feasibility of renal SN with lymphoscintigraphy and SPECT/CT reported non-visualization rates varying between 25 and 73% [11, 12]. Although inclusion criteria, timing of imaging, imaging, and mapping techniques were comparable, distinct methods for radiotracer injection were used and also radiotracer dose, imaged area, and modalities for SN detection varied (Additional file 1: Table S1). A few factors of Sherif et al.’s group that could theoretically have lowered their visualization rate were low volume of injected radiotracer and imaging only the abdominal area, whereas our group included thoracic lymphoscintigraphy, where 20% of SNs were found. Although, Sherif et al. also used blue dye intraoperatively, they detected only 12.5% of SN with this technique, which is in line with earlier reported significantly lower sensitivity of blue dye (50–70%) compared to radiotracer [12, 20]. Today, our study has the largest renal tumor SLNB cohort with 73 patients presenting 37% of non-visualization on scintigraphy and SPECT/CT imaging with intratumoral injection. In the study of Sherif et al. [12] consisting of 13 patients, the radioisotope was injected peritumorally. Non-visualization on scintigraphy and SPECT/CT was reported in 8 out of 11 (73%) of cases. They detected most of the SNs using a γ-probe, whereas in our study most of the SNs were visualized by scintigraphy combined with SPECT/CT, and the minority was detected with γ-probe only (*n* = 5 (18%)). Compared to SLNB in other primary tumor sites, which have scintigraphy and SPECT/CT visualization rates of 82–100%, SLN visualization in renal tumors is lower [9, 21].

It has been suggested in other tumor types that non-visualization of lymphatic drainage can be related to patient age, BMI, size of the tumor, high nodal tumor load, neoadjuvant therapy, occlusion of lymphatics, radioisotope type, dose and volume, and lymphovascular invasion [9, 14–16, 22]. Including all the possible factors (Table 2), we acknowledged some specific aspects that might be associated with non-visualization regarding kidney physiology. Due to high vascularity of the kidney and vascular tumors, inappropriate radiotracer injection into a highly vascularized area of the renal tumor may cause a wash-out phenomenon which might be related to non-visualization. However, our results were not conclusive about the association between non-visualization and wash-out phenomena in multivariable analysis.

The only aspect which demonstrated association with non-visualization in our group was age with a tendency toward having more non-visualization with older age. Age has been associated with false negative rate in melanoma and breast cancer patients and it has been speculated that it may be caused by degeneration of the lymphatic system resulting in decreased rate of lymph flow with increasing age or secondary to variable or sluggish lymphatics in older patients [14, 23–25]. In fact, change in lymphatic function of SNs in older patients and lymphatic aging have been demonstrated earlier [14, 25]. This has been shown by decline in radiotracer transit with older age in melanoma patients with intraoperative lower counts of radioactivity measured with a γ-probe. In addition, there has also been association between age and lower number of metastatic SNs which further suggests an alteration of lymphatic function with aging [14]. However, due to lacking records of radioactivity count measurements and low number of SN metastatic cases, further analysis to substantiate an association in our cohort is unavailable.

Other accepted determinants that have shown association with non-visualization in different tumor types are tumor location in the organ and method of injection (intratumorally, peritumorally) [26–28]. Comparison of intra-and peritumoral hybrid isotope injection in prostate cancer was studied recently, showing more non-visualization with intratumoral injection than peritumoral; however, more pN positive patients were detected with intratumoral method [26]. Detection of SNs has also been better with periareolar compared to intratumoral radiotracer injection in breast cancer [29]. However, considering the kidney’s high vascularity, these tumor-bearing organs are not comparable and in fact injecting radiotracer into both the kidney and tumor could cause substantial radiotracer wash-out. Aspects of injection, e.g., injection site, number of depots, etc., were not associated with non-visualization in our study; however, it has been described that most of the lymphatics in kidney cancers with sinus vein invasion locate peritumorally and less are seen within the tumors [30]. This would suggest that Sherif et al. with peritumoral injection [12] should have had higher rate of visualization; however, they detected most of the SNs by γ-probe only and non-visualization rate with SPECT/CT was even higher than in our series.

Another important consideration regarding radiotracer injection is tumor location in the kidney (anterior, posterior, upper, intermedial, lower pole). Even though our results did not demonstrate difference between the tumor location and visualization, ultrasound-guided intratumoral injection can be technically challenging especially for anterior location. In addition, dynamics of the kidney caused by breathing can challenge the precision of the injection.

Non-visualization could also be related to lymphatic drainage directly into the thoracic duct without interfering retroperitoneal lymph nodes, whereas posterior lumbar lymphatics are more inclined to this route [5, 13, 30–32]. Thoracic duct might be also the path for a lympho-venous connection and subsequent hematogenic metastases mainly into lungs which is one of the most common metastatic sites in renal cancer and may occur without concurrent retroperitoneal lymph node metastatic involvement. An indication for the alternative drainage from the posterior lumbar lymphatics directly into the thoracic duct would be an association of non-visualization with posterior radiotracer depots, multiple injections, or larger tumors, because in all these occasions probability of radiotracer drainage into the posterior lumbar lymphatics are theoretically higher [13]. However, due to methodology used, we were not able to confirm this in our study. We were also unable to confirm the route on imaging in the cases with non-visualization because scintigraphy and SPECT/CT imaging probably are not sensitive enough to detect minimal amounts of radioactivity in the thoracic duct.

In view of the experience from the studies performed by the two different groups [11, 12], it appears that the majority of SNs are visualized 2–4 h after tracer injection and in case of non-visualization, delayed imaging can be considered. Imaging should consist of planar scintigraphy of the trunk including the thoracic cavity because 20% of sentinel lymph nodes are visualized in this area [5], combined with SPECT/CT of the area of interest. Intraoperative SN detection rates with γ-probe and -camera were comparable to preoperative lymphoscintigraphy and SPECT/CT, indicating that timing between imaging and operation in our study was optimal. ^99m^Tc nanocolloid was used as a radiotracer in both studies; however, whether its combination with ICG could improve intraoperative detection rate with an impact to overall detection rate and also reduce the time spent on SN harvesting remains to be studied. Concerning the dosage, according to depot activity measurements, 225 MBq ^99m^Tc appears to be adequate. Injection site peri- or intratumorally is debatable; however, we believe that injection into periphery of the tumor avoiding necrotic areas could result in better distribution of the tracer because high vascularization of the kidneys may cause a wash-out phenomenon. Nevertheless, when high-risk tumors are studied, additional peritumoral injection can be considered due to a more peri- than intratumoral lymphatic distribution [30].

Our study has a number of limitations. We enrolled mostly low risk patients, who have low incidence of lymph node metastatic disease. In addition, the study was not primarily designed to detect non-visualization, thus it has the limitations inherent to any retrospective data analysis. Besides, this study is not able to explain a predominant cause for non-visualization. Finally, the number of patients is low which may have an effect on the overall statistical power in our study.

## Conclusion

Non-visualization with scintigraphy and SPECT/CT in renal tumors is high and older age is the only factor associated with non-visualization. Determining whether peritumoral radiotracer injection in high-risk tumors or hybrid radiotracer with ICG could improve the detection rate requires another prospective study with primarily detection rate as a primary endpoint.

## Additional file

Additional file 1: Comparison of techniques used in renal tumour SLNB studies. (XLSX 11 kb)

## Abbreviations

LN: Lymph node
LND: Lymph node dissection
NV: Non-visualization
RCC: Renal cancer
SLNB: Sentinel lymph node biopsy
SN: Sentinel lymph node
SPECT/CT: Single-photon emission computed tomography with computed tomography
